# Psoriasiform Dermatitis: From Pathogenesis to New Therapeutic Opportunities

**DOI:** 10.3390/life15071026

**Published:** 2025-06-27

**Authors:** Eugenia Veronica Di Brizzi, Stefano Caccavale, Caterina Mariarosaria Giorgio, Giuseppe Argenziano, Anna Balato

**Affiliations:** Dermatology Unit, University of Campania “L. Vanvitelli”, 80131 Naples, Italy; stefano.caccavale@unicampania.it (S.C.); caterinagiorgio80@gmail.com (C.M.G.); giuseppe.argenziano@unicampania.it (G.A.); anna.balato@unicampania.it (A.B.)

**Keywords:** psoriasiform dermatitis, inflammatory skin diseases, biologic treatments

## Abstract

Psoriasiform dermatitis refers to a spectrum of inflammatory skin disorders that resemble psoriasis both clinically and histologically. These conditions can occur idiopathically or as paradoxical reactions to biologic or targeted therapies, particularly in patients with atopic or autoimmune backgrounds. Histologic features often include acanthosis, parakeratosis, and lymphocytic infiltrates, but without the full molecular signature of classical psoriasis. This review provides an overview of psoriasiform dermatitis with a focus on its clinical presentation, differential diagnosis, and the immune pathways involved. Drug-induced forms, especially those triggered by anti-TNF agents, IL-4/IL-13 blockers, and JAK inhibitors, are highlighted due to their growing clinical relevance. We also summarize the main topical and systemic treatments, including corticosteroids, calcineurin inhibitors, PDE4 inhibitors, and JAK-STAT- or IL-23-targeted therapies. A better understanding of psoriasiform dermatitis is crucial to improve diagnosis and to guide treatment, especially in complex or refractory cases.

## 1. Introduction

Psoriasiform dermatitis represents an increasingly relevant diagnostic and therapeutic challenge, due both to the complexity of its clinicopathologic features and to the growing incidence of cases secondary to immunomodulatory therapies. The term refers to a group of inflammatory dermatoses that clinically and histopathologically resemble psoriasis, characterized by features such as acanthosis, parakeratosis, and epidermal hyperplasia [1]. However, these conditions are distinct entities often triggered by external factors like medications, infections, or immune dysregulation [2]. Unlike classic psoriasis, psoriasiform dermatitis includes a variety of etiologies and pathways, making it a valuable model for exploring skin inflammation and its systemic implications [3]. Clinically, psoriasiform dermatitis presents a significant diagnostic challenge due to its phenotypic overlap with chronic eczema, true psoriasis, and even early cutaneous lymphomas. Misclassification can lead to delayed or inappropriate treatment. In recent years, an increasing number of psoriasiform eruptions have been observed as the paradoxical effects of targeted biologic therapies, particularly in patients treated for atopic dermatitis or inflammatory bowel disease [2]. Despite its clinical relevance, psoriasiform dermatitis remains underrecognized, with the literature often fragmented across case reports, experimental models, and disease-specific studies. Therefore, a comprehensive review is warranted to clarify its pathogenesis, clinical presentation, diagnostic approaches, and current and emerging therapeutic strategies.

Psoriasiform dermatitis is relatively uncommon, and its precise incidence and prevalence figures remain unavailable due to its heterogeneous presentation and frequent misdiagnosis as classic psoriasis or eczema [1]. Several exogenous and endogenous triggers have been identified. Common exogenous factors include medications such as β-blockers, lithium, synthetic antimalarials, NSAIDs, tetracyclines, and digoxin [4]. In recent years, drug-induced psoriasiform dermatitis has gained recognition, particularly in patients receiving biologic therapies and small molecule inhibitors for autoimmune and inflammatory diseases [2,5,6]. Notably, paradoxical psoriasiform eruptions have been described with anti-TNF agents [5], IL-4/IL-13 inhibitors like dupilumab [6,7], and JAK inhibitors [8,9]. Some reports suggest that up to 5–10% of patients treated with anti-TNF therapies for inflammatory bowel disease may develop psoriasiform skin lesions [5]. Understanding these triggers is essential for accurate diagnosis and appropriate therapeutic strategies.

This review aims to provide an updated synthesis of the immunological and molecular mechanisms underlying psoriasiform dermatitis, highlight the histological and dermoscopic features that distinguish it from classic psoriasis, and discuss both conventional and novel treatments currently being explored in preclinical and clinical settings.

## 2. Methods

**Figure 1:**
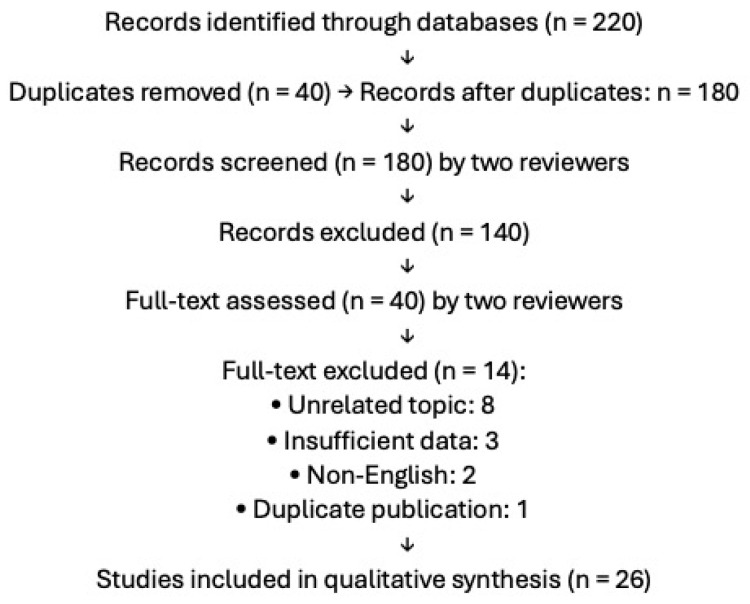
PRISMA flow diagram of the study selection process.

A comprehensive literature search was conducted using the PubMed, Scopus, Embase, and MEDLINE databases (up to March 2025). The search was performed using MeSH terms and free-text keywords combined with “psoriasiform dermatitis”, applying Boolean operators (e.g., AND). In particular, keywords related to the pathogenesis, clinical features, diagnosis, histology, and treatment of psoriasiform dermatitis were used. In addition, terms related to specific molecules and therapeutic approaches were included, such as “IL-17”, “IL-23”, “JAK inhibitors”, “apremilast”, “fingolimod”, “capsaicin”, “NO2-FAs”, and “dupilumab”. Only English-language articles were selected based on clinical relevance and methodological rigor, including reviews, meta-analyses, preclinical studies, case reports, and real-life data. The reference lists of the selected articles were also reviewed to identify additional relevant sources. This review is based solely on previously published data and does not include any studies conducted by the authors involving human or animal subjects. Titles and abstracts were screened independently by two reviewers (E.V.D.B. and S.C.); full texts were likewise independently assessed. Disagreements were resolved through consensus during discussion. Studies were excluded for the following reasons: unrelated topic (n = 8), insufficient data on psoriasiform dermatitis (n = 3), non-English language (n = 2), or duplicate publication (n = 1). The study selection process is illustrated in a PRISMA flow diagram (Figure 1), detailing the records identified, screened, excluded (with reasons), and included. In total, 26 studies were included in the qualitative synthesis.

## 3. Pathogenesis

The pathogenesis of psoriasiform dermatitis arises from a complex interaction between the immune system and skin-resident cells, mediated by a network of pro-inflammatory cytokines and molecular signals. A central element is the IL-23/IL-17/IL-22 axis, which drives keratinocyte activation, dermal inflammation, and pathological epidermal proliferation. IL-23, primarily produced by dendritic cells (DCs) activated through TLR7 receptors, promotes the differentiation of T cells into Th17 cells, which in turn release cytokines such as IL-17A and IL-22, amplifying skin inflammation. These processes were confirmed in experimental models, where the neutralization of IL-17 or IL-23 led to a significant reduction in skin lesions [10,11]. IL-36 cytokines also play a critical role, not only activating the NF-κB inflammatory pathway but also contributing to neutrophil recruitment and the production of chemokines like CXCL1 and CCL20. IL-36 acts in both an autocrine and paracrine fashion on skin-resident cells, such as keratinocytes and fibroblasts, further amplifying the inflammatory cycle and cellular proliferation [10]. More recently, the role of senescent CD4+ cells as critical mediators in IMQ-induced psoriasiform dermatitis has emerged. The elimination of these cells through specific inhibitors, such as BCL-2, has shown significant improvements in skin lesions, highlighting a novel therapeutic target [4]. Similarly, the blockade of the JAK-STAT pathway, despite its involvement in immune signaling, can worsen some forms of psoriasiform dermatitis, particularly when the condition overlaps with mycosis fungoides, a type of cutaneous lymphoma [12]. In addition to cytokines, recent studies have emphasized the role of metabolic regulators, such as LPCAT1, an enzyme that modulates glucose transport via GLUT3. LPCAT1 has been implicated in keratinocyte hyperproliferation and the promotion of skin inflammation, highlighting a connection between cellular metabolism and inflammatory pathogenesis [11]. Another study identified the protein PTX3 as a key regulator of macrophage polarization, exacerbating skin inflammation and suggesting further therapeutic targets [13]. Lastly, the effects of adiponectin deficiency in promoting psoriasiform-like skin inflammation have been explored. This deficit, observed in murine models, appears to worsen the activation of T cells and macrophages, amplifying inflammatory processes in the skin [14]. These findings highlight a complex network of cellular and molecular interactions underlying psoriasiform dermatitis, where immune, metabolic, and cellular factors cooperate to drive pathology. Emerging therapeutic approaches that specifically target pathways such as IL-36, IL-23/IL-17, or metabolic regulators offer new perspectives for treating this condition without compromising the broader immune system.

The immunomodulator Macrophage Migration Inhibitory Factor (MIF) has also been identified as a potential driver of psoriasiform dermatitis. Elevated in the skin and serum of psoriasis patients, MIF expression is further upregulated in mouse models of psoriasiform dermatitis induced by imiquimod and IL-23. MIF deficiency in these models resulted in reduced erythema, infiltration, and desquamation, along with decreased keratinocyte hyperproliferation and dermal angiogenesis. These findings suggest that MIF contributes to psoriasiform dermatitis pathogenesis through multiple mechanisms, particularly in IL-23-driven pathways, positioning it as a possible therapeutic target [15]. Recent research has revealed additional inflammatory mechanisms contributing to psoriasiform dermatitis. The overexpression of KLK6, a serine protease, has been linked to the development of psoriasiform dermatitis through the activation of PAR1-mediated pathways. This interaction plays a crucial role in amplifying inflammation and epidermal remodeling, suggesting KLK6/PAR1 signaling as a potential therapeutic target [16]. Furthermore, extracellular ATP has been identified as a key player in skin inflammation by activating the P2X7 receptor pathway. This activation leads to the stimulation of the IL-1β/NLRP3 inflammasome, which in turn promotes neutrophil recruitment and enhances inflammatory responses in psoriasiform dermatitis. Targeting the P2X7 receptor could therefore represent a novel approach for modulating disease severity and progression [17]. A variety of medications have been recognized as potential triggers for psoriasiform dermatitis. Among them, β-blockers, synthetic antimalarial drugs, non-steroidal anti-inflammatory drugs (NSAIDs), lithium, digoxin, and tetracycline antibiotics have been associated with both new-onset and the exacerbation of existing psoriasiform lesions, even in patients with no prior history of psoriasis [4]. Interestingly, biologic treatments used for inflammatory and autoimmune disorders have also been implicated in inducing psoriasiform lesions. Anti-TNF agents, despite being widely prescribed for psoriasis, have been linked to psoriasiform dermatitis as a paradoxical reaction [5]. Another biologic, Dupilumab, an IL-4Rα inhibitor primarily indicated for atopic dermatitis [6,7,18], has also been associated with the onset of psoriasiform dermatitis. This unexpected response is believed to result from an immune system shift from Th2 dominance to a Th1/Th17-driven inflammatory pathway, which is more closely linked to psoriasis pathogenesis. Such paradoxical reactions highlight the complexity of immune modulation in dermatologic conditions and emphasize the need for careful patient monitoring when prescribing biologic therapies [19,20]. Studies have also highlighted the role of genetic and molecular factors in psoriasiform dermatitis. Variations in the Itga11 gene, which encodes the α11 subunit of integrins, have been found to influence disease severity by modulating fibroblast function and immune responses. This genetic alteration affects skin inflammation and could serve as a potential biomarker for disease progression [21].

Additionally, proteins involved in skin barrier integrity, such as small proline-rich proteins (SPRR) and late cornified envelope (LCE) proteins, are significantly upregulated in psoriasiform dermatitis. These molecules, essential for epidermal differentiation and protective barrier function, are thought to contribute to the inflammatory process, further implicating epidermal integrity in disease pathogenesis [22]. Several factors can trigger or exacerbate psoriasiform dermatitis. A Western diet, characterized by high fat and sugar content, has been shown to promote the accumulation of IL-17A-producing γδ T cells, leading to skin inflammation even before significant weight gain occurs [2]. Moreover, certain medications, such as dupilumab used in atopic dermatitis, can induce psoriasiform erythema by shifting immune responses towards a Th1/Th17 predominance [2].

## 4. Clinical Features

Psoriasiform dermatitis can be difficult to distinguish from other conditions like eczema and psoriasis due to overlapping clinical and histopathological features. Histopathological examination is often necessary to confirm the diagnosis and guide treatment decisions [23]. Psoriasiform dermatitis commonly manifests as erythematous, scaly plaques resembling those seen in psoriasis [24,25]. These lesions may appear on various body sites, including the digits, back, scalp, abdomen, and extremities. The clinical presentation can vary in severity and distribution, often mimicking classical psoriasis, making differentiation between the two conditions challenging [2,26]. In pediatric cases, a distinctive form known as psoriasiform acral dermatitis has been documented [27,28]. This condition primarily affects the terminal phalanges, presenting as chronic dermatitis without nail dystrophy. While some cases appear linked to psoriasis, its classification as a distinct clinical entity remains under debate. Understanding its relationship with psoriasiform dermatitis and psoriasis is crucial for appropriate diagnosis and management.

## 5. Diagnosis

There is currently no universally accepted set of diagnostic criteria for psoriasiform dermatitis [1]. Diagnosis relies on an integrated approach that combines patient history (including identification of possible exogenous triggers such as drugs), thorough clinical examination, dermoscopic evaluation, and histopathological confirmation [2,29]. Clinically, psoriasiform dermatitis can resemble classic psoriasis, making differential diagnosis challenging [1,23]. Dermoscopy is a non-invasive tool that provides additional diagnostic clues by revealing specific vascular and scaling patterns [29,30,31]. It can help distinguish psoriasiform dermatitis from psoriasis and other similar conditions. Typical dermoscopic features include dotted or globular vessels arranged in a regular or irregular pattern and diffuse white scales; however, these findings are not pathognomonic [29]. Given the overlap with other inflammatory dermatoses, histopathological examination remains essential to support an accurate diagnosis and to exclude alternative conditions [1].

## 6. Histopathology

Histopathological examination of psoriasiform dermatitis reveals key features such as parakeratosis, acanthosis, and a superficial perivascular inflammatory infiltrate. These findings overlap significantly with those observed in psoriasis, complicating the diagnostic process. Despite the similarities, subtle histological differences and the clinical context may aid in distinguishing between these conditions [26,32].

Histologically, while psoriasis is characterized by Munro’s microabscesses, the thinning of the granular layer, and regular acanthosis, psoriasiform dermatitis tends to display a more heterogeneous pattern. Key histopathologic features include irregular acanthosis, parakeratosis, mixed perivascular inflammatory infiltrates, and occasionally eosinophils [2]. These findings are particularly evident in drug-induced forms or in paradoxical reactions to biologic therapies. The absence of a personal history of psoriasis and the identification of potential triggers (e.g., medications) are key in reaching the correct diagnosis.

## 7. Advanced Imaging

The use of technologies such as reflectance confocal microscopy or optical coherence tomography (LC-OCT) provides a detailed view of the skin’s microarchitecture [33,34], allowing for differentiation between inflammatory and non-inflammatory lesions. This technology can be particularly useful in monitoring treatment response and assessing lesion depth and extent.

## 8. Treatment Approaches

Psoriasiform dermatitis is a complex inflammatory skin condition that presents challenges in treatment due to its overlapping symptoms with psoriasis and other dermatological disorders. Advances in research have led to the exploration of various therapeutic approaches targeting key inflammatory mechanisms. The main systemic and topical options are summarized in Table 1.

### 8.1. Topical Therapies

While systemic treatments play a central role in managing moderate to severe psoriasiform dermatitis, topical therapies may be considered in milder cases or as adjuncts to systemic approaches. However, the current literature offers limited data on the efficacy of topical agents specifically in psoriasiform dermatitis. A case report described a patient with psoriasiform dermatitis who showed significant clinical improvement with a combination of topical corticosteroids and calcipotriol, although lesions recurred upon discontinuation of therapy [35]. Another report documented two patients who developed psoriasiform eruptions during dupilumab therapy; in both cases, lesions resolved following treatment with delgocitinib ointment, a topical JAK inhibitor [36]. Additionally, a preclinical study in a murine model demonstrated that the topical application of Entinostat, a class I histone deacetylase inhibitor, reduced inflammation and epidermal thickness in psoriasiform skin lesions, suggesting potential translational relevance [37].

### 8.2. Experimental and Non-Conventional Agents

Despite these promising individual reports, there is currently insufficient clinical evidence to establish standardized topical treatment protocols for psoriasiform dermatitis. Further investigations, including controlled trials and prospective case series, are needed to define the role of topical agents in this setting. One promising approach involves Electrophilic Nitro-Fatty Acids (NO2–FAs) [38], as explored in a study by Wang et al. Their research demonstrates that orally administered nitro-oleic acid (OA-NO2) can reduce both disease severity and inflammation in murine models by inhibiting the NF-κB and STAT3 pathways. OA-NO2 has been shown to decrease the production of inflammatory cytokines such as IL-1β, IL-6, and IL-17A while also limiting keratinocyte proliferation. Its primary mechanism of action appears to be the nitro-alkylation of STAT3, which reduces its phosphorylation and nuclear translocation, leading to beneficial effects on skin inflammation. These findings suggest that NO2–FAs could represent a promising therapeutic option for inflammatory skin diseases such as psoriasiform dermatitis. Another promising agent is fingolimod, a drug originally developed for multiple sclerosis. It has shown efficacy in psoriasiform dermatitis by sequestering IL-17A-producing γδ T cells within lymph nodes, preventing their migration to the skin. This mechanism ultimately leads to a reduction in inflammation and an improvement in symptoms [39]. Additionally, topical capsaicin has been investigated for its anti-inflammatory properties, particularly in modulating the IL-23/IL-17 signaling pathway. By downregulating cytokine production, capsaicin can help alleviate psoriasiform skin lesions and inflammation, offering a potential non-systemic therapeutic option [40].

### 8.3. JAK Inhibitors

Janus kinase (JAK) inhibitors represent a novel class of targeted therapies in dermatology, designed to modulate immune responses by interfering with the JAK-STAT signaling pathway. This pathway is crucial for cytokine-mediated processes involved in various inflammatory and autoimmune skin diseases. JAK inhibitors block specific JAK enzymes (JAK1, JAK2, JAK3, and TYK2), thereby reducing the activation of STAT proteins and, consequently, the expression of pro-inflammatory genes. This leads to a downregulation of key cytokines involved in skin inflammation, such as IL-4, IL-13, IL-17, IL-22, and IL-23 [41,42]. To ensure the safety of JAK inhibitors, it is essential to monitor infectious risk by screening for latent infections and assessing opportunistic infections. Cardiovascular health should be evaluated, particularly regarding thromboembolic and cardiovascular risks. Oncologic history must be considered, with ongoing surveillance for potential malignancies. Regular blood count assessments help detect any hematologic abnormalities, while lipid and metabolic profiles should be monitored for possible alterations [43]. JAK inhibitors have demonstrated effectiveness in managing psoriasiform dermatitis, especially in cases resistant to conventional treatments. With a favorable safety profile, they are being considered as a potential first-line therapeutic option [8]. Upadacitinib, a selective oral JAK 1 inhibitor currently approved for the management of moderate-to-severe atopic dermatitis, has demonstrated efficacy in treating psoriasiform skin eruptions, especially in patients who developed paradoxical reactions under biologic agents such as IL-17 or IL-23 inhibitors. A recent case report described a patient with severe psoriasiform dermatitis successfully managed with upadacitinib after the failure of multiple biologic therapies. In a multicenter case series, Shahriari et al. reported the successful use of upadacitinib in patients with either psoriasiform or spongiotic dermatitis. The authors observed significant clinical improvement in all treated patients, emphasizing the potential utility of this agent across a spectrum of chronic inflammatory dermatoses that share overlapping histologic features with psoriasis and eczema [44]. Similarly, a retrospective observational study by Salvi et al. described five patients with psoriasiform eczema and immune-mediated comorbidities, including ulcerative colitis and alopecia areata, who responded positively to treatment with upadacitinib. Notably, both skin lesions and associated systemic autoimmune manifestations improved during treatment, supporting the drug’s systemic immunomodulatory effect [9]. Additional evidence comes from case reports of psoriasiform dermatitis induced by dupilumab, a biologic agent used in atopic dermatitis. Patruno et al. and Cirone et al. both documented the resolution of dupilumab-induced psoriasiform eruptions following the initiation of upadacitinib, highlighting its value not only as a primary treatment but also as a rescue therapy in paradoxical inflammatory reactions [20,45]. Finally, Foggia et al. described the use of upadacitinib in patients with overlapping features of psoriasis and atopic dermatitis, reinforcing its clinical relevance in cases with mixed or ambiguous inflammatory patterns [46].

Similarly, Tofacitinib, a pan-JAK inhibitor, has been shown to reduce inflammation and improve skin lesions in inflammatory skin conditions with psoriasiform features, although evidence specific to psoriasiform dermatitis remains limited to case reports and off-label use. A notable case reported by Luo et al. described a patient with plaque psoriasis who developed paradoxical psoriasiform dermatitis while on secukinumab, an IL-17 inhibitor. The introduction of tofacitinib led to significant resolution of the skin lesions, suggesting its utility in managing adverse inflammatory reactions triggered by biologic therapies [47]. Further support comes from the review by Tegtmeyer et al., which explored the off-label use of tofacitinib in dermatology. The authors highlighted its effectiveness in a range of inflammatory dermatoses, including psoriasis, indicating a broader therapeutic potential in related conditions such as psoriasiform dermatitis [48]. Despite its clinical benefits, the safety profile of tofacitinib warrants careful consideration. A recent safety review on JAK inhibitors in dermatology emphasized the need for routine clinical and laboratory monitoring due to potential risks such as infections, thrombosis, and hematologic abnormalities [43]. Further evidence for the utility of JAK inhibitors in managing psoriasiform conditions comes from a recent retrospective study by Napolitano et al., which investigated the effectiveness of upadacitinib, baricitinib, and abrocitinib in adult patients with psoriasiform atopic dermatitis, a distinct clinical phenotype characterized by the coexistence of eczematous and psoriasiform lesions. Among 192 patients with moderate-to-severe atopic dermatitis treated with JAK inhibitors, 21 exhibited the psoriasiform variant. Remarkably, 95% of the patients achieved EASI-75 and 86% achieved EASI-90 by week 24. Significant improvements in pruritus and quality of life were also reported, and no adverse events led to treatment discontinuation [8].

### 8.4. PDE4 Inhibitors (Apremilast)

Apremilast is a selective phosphodiesterase 4 (PDE4) inhibitor approved for the treatment of plaque psoriasis and psoriatic arthritis. Its mechanism of action involves modulating inflammatory responses by increasing intracellular cyclic adenosine monophosphate (cAMP) levels, which in turn leads to reduced production of pro-inflammatory cytokines such as TNF-α and IL-17 [49]. A preclinical study evaluated apremilast in a murine model of imiquimod-induced psoriasiform dermatitis. The results showed that apremilast decreased interleukin-17 (IL-17) production and increased regulatory B and T cells in the spleen, suggesting a potential immunomodulatory effect in psoriasiform skin inflammation [50]. Case reports have described the successful use of apremilast in patients with psoriasiform dermatitis refractory to multiple systemic and biologic therapies. In one report, apremilast was effective in a patient with cancer-associated psoriasiform dermatitis after the failure of conventional treatments, suggesting it may represent a viable therapeutic option in complex or treatment-resistant cases [35].

### 8.5. Anti-IL-17

Anti-IL-17 inhibitors, including secukinumab, ixekizumab, brodalumab, and bimekizumab [51,52], are well-established therapeutic options in psoriasis, and their use has also been explored in selected cases of psoriasiform dermatitis. Notably, secukinumab has proven effective in the treatment of psoriasiform eruptions secondary to cancer immunotherapy, leading to resolution of skin lesions without compromising the antitumor response [53]. However, paradoxical adverse events associated with this drug class have also been reported. For example, atopic dermatitis-like eruptions have emerged in some patients treated with secukinumab, possibly due to a shift in the immune response toward a Th2 phenotype [54]. In addition, a case of drug-induced psoriasiform alopecia was described in a patient receiving ixekizumab [55] and a case series documented eczematous eruptions in psoriasis patients treated with anti-IL-17A agents, highlighting the need for close dermatologic monitoring during therapy [56]. These findings suggest that while anti-IL-17 drugs represent a promising therapeutic approach in psoriasiform dermatitis, they should be used with caution, with careful consideration of the patient’s immunological profile and potential for unexpected adverse reactions.

### 8.6. Anti-IL-23

Anti-IL-23, including guselkumab, tildrakizumab, and risankizumab, are biologic therapies approved for moderate-to-severe plaque psoriasis [24,25,57,58]. Although their efficacy in psoriasiform dermatitis is not yet well established, some preclinical studies and case reports suggest a potential benefit. In a murine model of imiquimod-induced psoriasiform dermatitis, IL-23 blockade with an anti-IL-23p19 antibody led to a significant reduction in skin inflammation, while promoting IL-10 expression and regulatory T-cell expansion, suggesting an immunomodulatory effect [59]. Another study showed that the monoclonal antibody IBI112, targeting IL-23p19, effectively neutralized IL-23 and consequently reduced IL-17 production, leading to improved skin lesions in psoriasis mouse models [60]. Despite these promising preclinical findings, the clinical literature specifically addressing the use of IL-23 inhibitors in psoriasiform dermatitis remains limited, and further controlled clinical trials are needed to better define their therapeutic role in this condition.

### 8.7. Anti-IL-4/IL-13

Currently, the literature regarding the use of dupilumab or tralokinumab in psoriasiform dermatitis is extremely limited. There are no clinical studies or case reports specifically documenting the therapeutic efficacy of these agents in this condition. However, a few reports describe the emergence of psoriasiform eruptions in patients treated with dupilumab for other diseases, particularly atopic dermatitis. One case described a 17-year-old male with atopic dermatitis who developed psoriasiform facial lesions after 16 months of treatment with dupilumab; the eruption improved following drug discontinuation and topical therapy [61]. Another report documented de novo psoriasis in a patient receiving dupilumab for atopic dermatitis, suggesting a possible shift in immune response from a Th2 to a Th1/Th17 phenotype [62]. Despite these observations, there is currently no evidence supporting the direct use or efficacy of dupilumab or tralokinumab in psoriasiform dermatitis, and further research is needed to explore their potential therapeutic applications in this setting.

## 9. Conclusions

Psoriasiform dermatitis represents a heterogeneous group of inflammatory skin conditions that share clinical and histopathological similarities with psoriasis but differ in etiology, pathogenesis, and therapeutic response. Recent advances have shed light on the complex immunologic and molecular mechanisms underlying this disorder, including the pivotal roles of the IL-23/IL-17 axis, IL-36 signaling, metabolic dysregulation, and senescent immune cells. The therapeutic landscape for psoriasiform dermatitis is evolving, with emerging data on the efficacy of targeted agents such as JAK inhibitors, PDE4 inhibitors, and biologics directed against IL-17 and IL-23. While some of these treatments have shown promise in case reports and preclinical studies, robust clinical evidence remains limited. Topical therapies may offer benefits in mild or localized forms, although standardized protocols are lacking.

Overall, the management of psoriasiform dermatitis requires a personalized and flexible approach, guided by disease severity, clinical phenotype, and underlying triggers. Further clinical trials and mechanistic studies are warranted to better define treatment strategies, identify predictive biomarkers, and ensure the safe and effective use of novel therapies in this complex and underrecognized condition.

## Figures and Tables

**Table 1:** Main systemic and topical treatments for psoriasiform dermatitis classified by target or pharmacologic class.

Class	Agents
Topical therapies	Corticosteroids, Vitamin D analogs (Calcipotriol), Retinoids, Calcineurin inhibitors (Tacrolimus, Pimecrolimus)
JAK/STAT inhibitors	Delgocitinib (topical), Upadacitinib, Baricitinib, Abrocitinib, Tofacitinib, Gusacitinib
IL-4/IL-13 blockade	Dupilumab (IL-4/IL-13), Tralokinumab (IL-13)
PDE4 inhibitors	Apremilast, AFX5931
Retinoids (systemic)	Alitretinoin
IL-17 inhibitors	Secukinumab, Ixekizumab, Brodalumab, Bimekizumab
IL-23 inhibitors	Guselkumab, Risankizumab, Tildrakizumab
Experimental agents	NO2-FAs, Fingolimod, Capsaicin
Systemic steroids	Prednisone, Methylprednisolone
